# Effect of lumbar epidural steroid injection on neuropathic pain: a prospective observational study

**DOI:** 10.3934/Neuroscience.2022003

**Published:** 2022-01-18

**Authors:** Chan Hong Park, Sang Ho Lee

**Affiliations:** 1 Department of Anesthesiology and Pain Medicine, Daegu Wooridul Spine Hospital of Daegu, Daegu, South Korea; 2 Department of Neurosurgery, Chungdam Wooridul Spine Hospital, Seoul, South Korea

**Keywords:** neuropathic pain, epidural, steroid

## Abstract

**Background:**

Low back pain (LBP) is caused by disc herniation, spinal stenosis, facet syndrome or etc. This LBP could be either nociceptive or neuropathic pain (NP). In addition, these neuropathic pain is a major contributor to chronic low back pain. It is already known that lumbar epidural steroid injection (ESI) is effective for low back pain, but no study has assessed both nociceptive and neuropathic pain separately. This study investigated whether neuropathic or nociceptive pain was better improved after an epidural steroid injection.

**Methods:**

This was a prospective study. Patients were classified according to the pre-procedure painDETECT questionnaire (PD-Q) score. If the PD-Q score was ≤12, it was considered as nociceptive pain, and it the PD-Q was ≥19, it was considered NP. The patients were given a transforaminal (TF) or interlaminar (IL) epidural steroid injection (ESI). The PD-Q was filled out by each patient prior to the ESI (baseline), and again at 4 weeks after the ESI. Outcomes was assessed using a numerical rating scale (NRS) score, short form McGill Pain Questionnaire (MPQ), and revised Oswestry Back Disability Index (ODI) at 1 month later.

**Results:**

A total of 114 patients were enrolled and of these, 54 patients with a PD-Q score of ≤12 were classified into the nociceptive pain, and 60 patients with a PD-Q score ≥19 were classified into the neuropathic pain group. At 1 month after treatment, both groups had significantly lower than improved their mean NRS score. Not withstanding these improvements and difference between NRS, the differences in MPQ and ODI after treatment between the groups (nociceptive vs. neuropathic) not significant. After the procedure (TF-ESI or IL-ESI), the patients in group 1 (PD-Q score ≤12, n = 54) had no change in their PD-Q score. Among the patients in group 2 (pre-treatment PD-Q score ≥19, n = 41), 13 patients moved to a PD-Q score <12 and 15 patients had a PD-Q score of 13–18.

**Conclusion:**

For the short-term relief of neuropathic pain, ESI was effective for both nociceptive and neuropathic pain, therefore ESI could be treat the try neuropathic pain component in patients with low back pain.

## 1. Introduction

Chronic low back pain (LBP) is often caused by lumbar disc herniation, spinal stenosis, and degenerative spondylolisthesis. When the compression of the nerve root occurs, patients experience pain due to a strong inflammatory reaction, therefore epidural injection of corticosteroids appears to be a reasonable treatment option [1]–[4].

An epidural steroid injection (ESI) is often used to treat neuropathic pain (NP) [5],[6]. The pain mechanism of nociceptive pain and neuropathic pain are different.

Neuropathic pain can be caused by actual neural compression or neural damage, but nociceptive pain is reaction to painful stimuli [7]. For patients with failed back surgery syndrome with prominent radicular symptoms, ESI is a reasonable treatment option for both clinicians and the patients when the patients have failed to respond to less invasive treatments but are not ready to consider more invasive treatments, such as spinal cord stimulation [8]. Steroids including 80 mg methylprednisolone are efficient at alleviating a patient's overall pain, and both interlaminar (IL) and transforaminal (TF) epidural steroid injection (ESI) can reduce the neuropathic component in patients with chronic lumbar radicular pain [6]. In addition, an 80 mg triamcinolone TFESI has been reported to provide a substantial improvement in patients' pain and neuropathic pain and quality of sleep, but had no effect on patient the quality of life [5].

However, no study has examined the proportion of pain relief that could be attributed to treating the neuropathic pain component. The current study examined which component of pain (neuropathic or nociceptive) was most improved after an epidural steroid injection.

## 2. Methods

The protocol for this prospective observational study was approved by the Ethics Review Committee.

The study inclusion criteria were as follows: aged 18–80 years; lumbar radicular pain/or low back pain that had not responded to traditional treatments (pharmacotherapy and physical therapy) within the previous 4 weeks; confirmed the pathology of the lumbar radicular pain confirmed by magnetic resonance imaging; and absence of a remarkable motor deficit and bowel/urinary incontinence. The study exclusion criteria were as follows: under 18 or over 80 years of age; low back pain only; diabetes; a progressive neurological disorder; history of allergic reactions to local anesthesia, opiates, contrast agents or steroids; history of opioid abuse or currently on long-term opioid treatment.

The interlaminar epidural steroid injection (IL-ESI) was performed with a 20-gauge Touhy needle and 5 mg of dexamethasone mixed with 5 mL of 0.5% lidocaine. The transforaminal epidural steroid injection (TF-ESI) was performed with a 22-gauge needle and a solution of 5 mg dexamethasone in 3 mL of 0.5% lidocaine. The patients did not receive any anticonvulsants or antidepressants during the observation period, but during a 2 week period after the procedures, all subjects were received NSAID, and muscle relaxant. Patients who experienced breakthrough pain received 50 mg tramadol as a rescue medication.

The painDETECT questionnaire (PD-Q) was filled out by each patient prior to the ESI (baseline), and again at 4 weeks after the ESI. If the PD-Q score was ≤12, it was considered nociceptive pain, and PD-Q was ≥19, it was considered as NP.

Outcomes were assessed using the numerical rating scale (NRS) score, short form McGill Pain Questionnaire (MPQ), and revised Oswesry Back Disability Index (ODI) measured at pre-treatment and 1 month later.

All data were analyzed using IBM SPSS Statistics for Windows, Version 23.0 (IBM; Armonk, NY) The statistical analyses were performed using basic methods of descriptive statistics. The Student's t-test or Mann Whitney U test was used for comparing mean values. P-values ≤ 0.05 were considered statistically significant. The sample size of 114 was specified in advance to provide 90% power to detect a difference.

## 3. Results

The study enrolled a total of 114 patients. Based on the pre-procedure PD-Q score, 54 patients had a PD-Q score of ≤12 (nociceptive pain) and 60 patients had a PD-Q score ≥19 (Table 1).

The mean values of NRS, MPQ, and ODI before treatment were not significantly different between the two groups (Table 2). At 1 month after treatment, both groups had significantly lower than improved their mean NRS score (P = 0.000) (Table 2). Notwithstanding these improvements and the difference in the NRS score, the differences in MPQ and ODI after treatment between the groups (nociceptive vs. neuropathic) were not significant (Table 2). After the procedure (TF-ESI or IL-ESI), the patients in group 1 (PD-Q score ≤12, n = 54) had no change in their PD-Q score. Among the patients in group 2 (pre-treatment PD-Q score ≥19, n = 41), 13 patients moved to a PD-Q score <12 and 15 patients had a PD-Q score of 13–18 (Table 3).

**Table 1.** Patient's characteristics.

N = 114	Nociceptive (n = 54) group	Neuropathic (n = 60) group
PD-Q	≤ 12	19 ≤
Gender (M : F)	30 : 24	29 : 31
Age (yrs)	51.1 ± 14.2	53.4 ± 13.5
Diagnosis		
DDD	12	10
HIVD	26	30
Stenosis	14	14
Spondylolisthesis	2	6
Level		
L3–4	4	10
L4–5	42	40
L5–S1	8	10

Note: PD-Q: painDETECT questionnaire, DDD: degenerative disc disease, HIVD: herniated intervertebral disc.

**Table 2.** Values of the numeric raring scale score (NRS), McGill Pain Questionnaire and Oswestry Disability Index (ODI) before and 1 month after epidural steroid injection treatment in patients with low back pain.

	Baseline	1 month	P value
*Nociceptive (n = 54)*			
NRS	7.5 ± 0.5	3.9 ± 2.6	0.000
McGill			
Sensory	16.7 ± 6.0	16.8 ± 6.2	0.383
Affective	5.0 ± 2.3	4.8 ± 2.1	0.159
ODI	57.7 ± 4.9	58.4 ± 5.7	0.152
*Neuropathic (n = 60)*			
NRS	7.5 ± 0.8	4.2 ± 1.8	0.000
McGill			
Sensory	14.8 ± 5.6	14.7 ± 5.6	0.339
Affective	5.2 ± 1.7	4.9 ± 1.4	0.306
ODI	54.0 ± 5.0	55.0 ± 3.8	0.152

**Table 3.** Change in the patient's painDETECT questionnaire (PDQ) score after epidural steroid injection (ESI).

	Pre-ESI PDQ		Post-ESI PDQ		
	PDQ ≤ 12	19 ≤ PDQ	PDQ ≤ 12	12 < PDQ < 19	19 ≤ PDQ
Nociceptive pain (n = 54)	54	0	54	0	0
Neuropathic pain (n = 60)	0	60	13	15	32

## 4. Discussion

In the present study, there was no difference in pain reduction between the nociceptive pain and neuropathic pains after ESI was not significant, and we found that the difference in providing relief for neuropathic pain between triamcinolone and dexamethasone was not significant.

Our findings are consistent with those from a previous studies [5],[6], in which ESI (80 mg of triamcinolone plus 3 mL of 0.5% bupivacaine; 3 months follow up) provided a substantial improvement in patient pain levels, neuropathic pain levels, and quality of sleep, but had no effect on quality of life [5].

Neuropathic pain is caused by damage to the afferent peripheral or central nervous system. Additionally, neuropathic pain may be caused by mechanical compression of nerve roots (mechanical neuropathic root pain), lesions of nociceptive sprouts within a degenerated disc (local neuropathic pain), or by the actions of inflammatory mediators such as chemokines and cytokines, which can originate from a degenerative disc even in the absence of mechanical stress ithat causes nflammatory neuropathic root pain [6].

An epidural steroid reduces inflammation [9]–[11], which leads to reduced in pain.

Generally, triamcinolone (particulate steroid) or dexamethasone (non-particulate steroid) are used for ESI. There has been some debate as to which steroid is superior. Patients who received transforaminal ESI with triamcinolone reported more frequent pain relief of greater than 50% at short-term follow-up compared with those who received betamethasone [12],[13]. However, another study of patients with cervical radiculopathy reported that triamcinolone (40 mg) and dexamethasone (15 mg) produced similar benefits as measured by the patients' self-reported pain scores [14]. In our study, the neuropathic component was concerted to nociceptive or intermediate in neuropathic group. We postulated that a local anesthetic or steroid may be effective for neuropathic pain.

Triamcinolone and dexamethasone have different durations of activity action and anti-inflammatory potency. Dexamethasone acts rapidly and long duration of action with greater anti-inflammatory potency than triamcinolone [15]. In addition, dexamethasone has particulate size <5 µm and have lowest density and lower tendency for aggregation [16] than triamcinolone. Triamcinolone has an intermediate action duration and anti-inflammatory potency. Triamcinolone consists of particles, or that can form aggregates that are larger than red blood cells [16].

We postulated that patients in the nociceptive pain group would obtain more pain relief than those in the neuropathic pain group. However, our results showed that the difference in pain reduction was not significant. Although, the etiology of neuropathic pain is highly variable when compared with the etiology of nociceptive pain, some drugs are only effective for treating a single specific type of pain (either nociceptive or neuropathic pain).

There has been controversy on the safety of ESI [17]. Brain or spinal cord infarctions have been reported after an ESI [17],[18]. Although the risk of complication after ESI into lumbar region was smaller than the risk after cervical or thoracic ESI, serious complications such as neural infarctions have occurred [17]. Inadvertent intra-arterial (radicular artery) injection of a particulate steroid can create an embolu that can lead to infarction [19]–[23].

The study was limited by the fact that patient outcomes were assessed only by the patients' self-reported pain scores and that short follow-up period was short.

In conclusion, ESI was effective in both nociceptive and neuropathic pain, therefore ESI could be try to treat neuropathic pain component in patients with chronic low back pain.
